# Exposures associated with the onset of Kawasaki disease in infancy from the Japan Environment and Children’s Study

**DOI:** 10.1038/s41598-021-92669-z

**Published:** 2021-06-25

**Authors:** Sayaka Fukuda, Shiro Tanaka, Chihiro Kawakami, Tohru Kobayashi, Shuichi Ito, the Japan Environment and Children’s Study (JECS) Group, Michihiro Kamijima, Shin Yamazaki, Yukihiro Ohya, Reiko Kishi, Nobuo Yaegashi, Koichi Hashimoto, Chisato Mori, Zentaro Yamagata, Hidekuni Inadera, Takeo Nakayama, Hiroyasu Iso, Masayuki Shima, Youichi Kurozawa, Narufumi Suganuma, Koichi Kusuhara, Takahiko Katoh

**Affiliations:** 1 Department of Pediatrics, Graduate School of Medicine, Yokohama City University, 3-9 Fukuura, Kanazawa-ku, Yokohama, Kanagawa 236-0004 Japan; 2 Department of Pediatrics, Saiseikai Yokohamashi Tobu Hospital, Yokohama, Japan; 3 Department of Clinical Biostatistics/Clinical Biostatistics Course, Graduate School of Medicine, Kyoto University, Kyoto, Japan; 4 Department of Data Science, Clinical Research Center, Hospital, National Center for Child Health and Development, Tokyo, Japan; 5 Graduate School of Medical Sciences Department of Occupational and Environmental Health, Nagoya City University, 1 Kawasumi, Mizuho‑cho, Mizuho‑ku, Nagoya, Aichi 467‑8601 Japan; 6 National Institute for Environmental Studies, Tsukuba, Japan; 7 National Center for Child Health and Development, Tokyo, Japan; 8 Hokkaido University, Sapporo, Japan; 9 Tohoku University, Sendai, Japan; 10 Fukushima Medical University, Fukushima, Japan; 11 Chiba University, Chiba, Japan; 12 University of Yamanashi, Chuo, Japan; 13 University of Toyama, Toyama, Japan; 14 Kyoto University, Kyoto, Japan; 15 Osaka University, Suita, Japan; 16 Hyogo College of Medicine, Nishinomiya, Japan; 17 Tottori University, Yonago, Japan; 18 Kochi University, Nankoku, Japan; 19 University of Occupational and Environmental Health, Kitakyushu, Japan; 20 Kumamoto University, Kumamoto, Japan

**Keywords:** Vasculitis syndromes, Epidemiology

## Abstract

Kawasaki disease (KD) is an acute systemic vasculitis that mainly affects infants and young children. The etiology of KD has been discussed for several decades; however, no reproducible risk factors have yet been proven. We used the Japan Environment and Children’s Study data to explore the association between the causal effects of exposure during the fetal and neonatal periods and KD onset. The Japan Environment and Children’s Study, a nationwide birth cohort study, has followed approximately 100,000 children since 2011. We obtained data on exposures and outcomes from the first trimester to 12 months after birth. Finally, we included 90,486 children who were followed for 12 months. Among them, 343 children developed KD. Multivariate logistic regression revealed that insufficient intake of folic acid during pregnancy (odds ratio [OR], 1.37; 95% CI 1.08–1.74), maternal thyroid disease during pregnancy (OR, 2.03; 95% CI 1.04–3.94), and presence of siblings (OR, 1.33; 95% CI 1.06–1.67) were associated with KD onset in infancy. In this study, we identified three exposures as risk factors for KD. Further well-designed studies are needed to confirm a causal relationship between these exposures and KD onset.

## Introduction

Kawasaki disease (KD) is an acute systemic vasculitis that mainly affects infants and young children^1^. If untreated, approximately 25% of patients with KD develop coronary artery aneurysm. KD is the leading cause of pediatric acquired heart disease in developed countries^2^. Patients with KD who develop coronary artery aneurysm are at risk of ischemic heart disease and require prolonged anticoagulation therapy.

KD is thought to be triggered by infection or unknown exposures in children with genetic predispositions and/or of specific ethnicities, including East Asians^3,4^. KD also has a high incidence in infants and toddlers in whom innate immunity is more predominant than acquired immunity, although the true etiology of KD in these groups remains unknown. On the basis of these epidemiological features of KD, infectious agents (bacteria, including group A streptococci, *Staphylococcus aureus*, *Yersinia pseudotuberculosis*, and viruses, including respiratory viruses, Epstein–Barr virus, and human adenovirus) are associated with KD onset^5–10^. Recently, multisystem inflammatory syndrome in children, which develops after SARS-CoV-2 infection and has overlapping symptoms with KD, has been reported^11,12^. Multisystem inflammatory syndrome in children may provide novel immunological insights into the pathogenesis of KD^13^. Further studies are expected in the future. There are also several reports that patients’ microbiome may stimulate the innate immune system and lead to KD development^14,15^.

Some risk factors for KD, such as using rug shampoo, older maternal age, and nutritional exposures (e.g., a shorter duration of breastfeeding and supplementation with vitamin D or soy isoflavone), identified in small-scale retrospective studies with limited target areas and participants. have not been reproducible in other studies^16–19^. Yorifuji et al.^20^ reported that early childhood exposure to maternal smoking might increase the risk of KD, and Fujiwara et al.^21^ reported that a higher socioeconomic status might increase the risk of KD. The findings of both studies used data from a large-scale longitudinal Japanese cohort study^22^. Belkaibech et al.^23^ subsequently reported an association between maternal autoimmune disease and KD onset in a large retrospective Canadian cohort. Unfortunately, these studies did not prospectively investigate fetal exposure. None of the risk factors reported in these studies have been verified in large-scale prospective studies.

The Japan Environment and Children’s Study (JECS) is a large prospective Japanese birth cohort study of approximately 100,000 children^24,25^. Its purpose is to prospectively evaluate the effects of exposures during the fetal stage and early childhood on pediatric health and development. The age at KD onset in Japan shows a monomorphic distribution with a peak at 9–11 months^26^. Although various factors have been implicated in the onset of KD, we hypothesized that exposures during the fetal and neonatal periods are associated with KD onset in infancy. We used data from the JECS to explore the association between the causal effects of exposures during the fetal and neonatal periods and KD onset. Our goal was to identify prenatal and perinatal exposures associated with early onset of KD using the JECS database.

## Results

**Figure 1:**
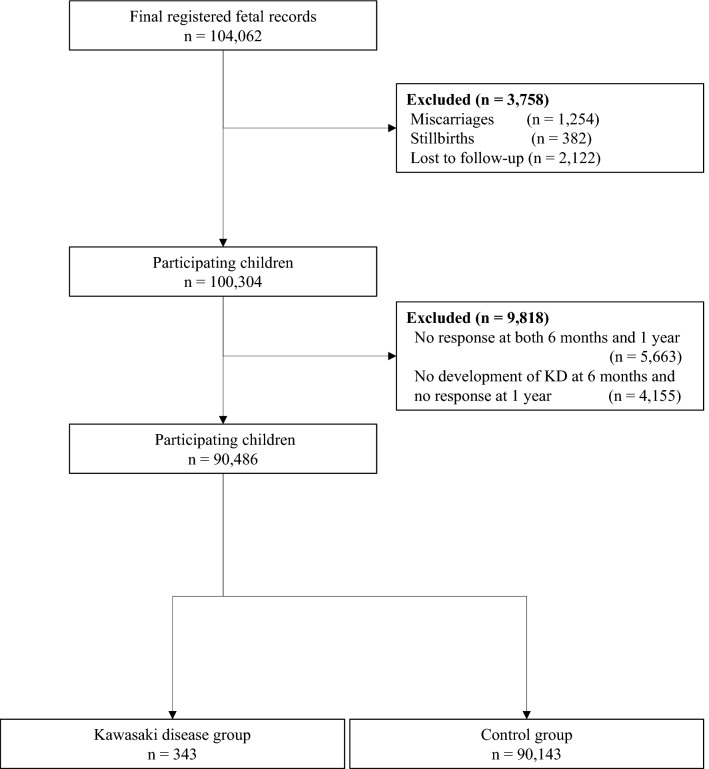
Participant flowchart.

Figure 1 shows a flowchart of participant selection in our study. In total, 104,062 fetal records were registered during the study period. Finally, we included 90,486 children as the analysis population. Self-administered questionnaires at 12 months reported KD development in 343 of 90,486 children (0.38%; 95% CI 0.34–0.42%).

Of 343 children with KD, detailed clinical information for 312 children (91.0%) was obtained from the KD-specific questionnaire. The mean age at KD onset was 6.9 ± 3.0 months (median, 7 months; range, 0–13 months). A total of 221 children (70.8%) fulfilled ≥ 5 major symptoms, and 91 children (29.2%) had incomplete KD. The parents of three (1.0%) children and the siblings of five children (1.6%) had a history of KD. Intravenous immunoglobulin was administered to 291 children (93.3%), although 47 (16.2%) had refractory disease. Nineteen (6.1%) children had complications in the form of coronary artery dilation or aneurysm during the acute phase of KD.

Table 1 shows the parental characteristics of participants. Neither a maternal nor paternal history of KD was significantly associated with KD onset in these children. However, a paternal history of allergic disease was significantly associated with KD onset. However, the paternal medical history was missing for approximately 50% of participants. Other maternal physical characteristics and socioeconomic status were not significantly associated with KD onset.

**Table 1:** Parental characteristics of the study population.

Variables	KD group (n = 343)	Control group (n = 90,143)	Odds ratio	95% CI	P value
**Maternal pre-pregnancy physical characteristics**					
Height, cm	158.6 ± 5.2	158.1 ± 5.3	0.85	0.70–1.04	0.112
	158.0 (143.0–173.0)	158.0 (130.0–183.0)			
Weight before pregnancy, kg	53.0 ± 8.9	53.1 ± 8.8	1.01	0.89–1.14	0.888
	51.0 (39.0–95.0)	52.0 (29.0–127.6)			
BMI before pregnancy, kg/m^2^	21.1 ± 3.4	21.2 ± 3.3	0.99	0.95–1.02	0.411
	20.1 (15.4–41.7)	20.5 (13.2–52.8)			
**Maternal medical history**					
KD	1/341 (0.3)	388/89,595 (0.4)	0.68	0.09–4.83	0.696
Allergic disease	182/341 (53.4)	49,748/89,595 (55.5)	0.92	0.74–1.13	0.425
Neurological/psychiatric disease	48/341 (14.1)	12,196/89,595 (13.6)	1.04	0.77–1.41	0.803
Diabetes mellitus	4/341 (1.2)	842/89,595 (0.9)	1.25	0.47–3.36	0.657
Cancer	7/341 (2.1)	960/89,595 (1.1)	1.94	0.91–4.10	0.085
**Paternal medical history**					
KD	2/150 (1.3)	201/46,635 (0.4)	3.12	0.77–12.69	0.112
Allergic disease	85/150 (56.7)	21,633/46,635 (46.4)	1.51	1.09–2.09	0.012
**Parental socioeconomic status**					
Maternal education					
Junior high or high school graduate	118/340 (34.7)	310,86/89,023 (34.9)	Reference	–	0.235*
Technical junior college or technical/vocational college graduate or associate degree	130/340 (38.2)	37,952/89,023 (42.6)	0.90	0.70–1.16	
Bachelor’s degree or beyond	92/340 (27.1)	19,985/89,023 (22.4)	1.21	0.92–1.59	
Paternal education					
Junior high or high school graduate	142/337 (42.1)	38,067/88,530 (43.0)	Reference	–	0.966*
Technical junior college or technical/vocational college graduate or associate degree	83/337 (24.6)	20,094/88,530 (22.7)	1.11	0.84–1.45	
Bachelor’s degree or beyond	112/337 (33.2)	30,369/88,530 (34.3)	0.99	0.77–1.27	
Household income					
< 2 million JPY	17/319 (5.3)	4,383/83,424 (5.3)	Reference	–	0.923*
2 to < 4 million JPY	100/319 (31.3)	28,474/83,424 (34.1)	0.91	0.54–1.52	
4 to < 6 million JPY	123/319 (38.6)	27,850/83,424 (33.4.)	1.14	0.69–1.89	
6 to < 10 million JPY	64/319 (20.1)	19,092/83,424 (22.9)	0.86	0.51–1.48	
≥ 10 million JPY	15/319 (4.7)	3,625/83,424 (4.3)	1.07	0.53–2.14	

Data are presented as n (%), mean ± standard deviation, or median (range).

*BMI* body mass index, *KD* Kawasaki disease, *JPY* Japanese yen, *95% CI* 95% confidence interval.

*P-value for trend.

Tables 2 and 3 show the maternal prenatal findings and perinatal backgrounds of participants. Maternal complications of thyroid disease during pregnancy were significantly associated with KD onset in children. However, only nine mothers (2.6%) in the KD group had thyroid disease. Presence of siblings of the affected child was significantly associated with KD onset. The frequency of folic acid supplementation of mothers during pregnancy was also significantly associated with KD onset.

**Table 2:** Maternal prenatal findings of the study population.

Variables	KD group (n = 343)	Control group (n = 90,143)	Odds ratio	95% CI	P value
Spontaneous pregnancy	311/343 (90.7)	83,336/90,102 (92.5)	0.79	0.55–1.14	0.203
**Pregnancy complications**					
Thyroid disease	9/343 (2.6)	1,213/89,952 (1.3)	1.97	1.01–3.83	0.045
Diabetes mellitus	2/343 (0.6)	961/89,952 (1.1)	0.54	0.14–2.18	0.390
**Contents of meals in second and third trimesters**					
Beans, g/day	59 ± 143	54 ± 76	1.00	1.00–1.00	0.229
	36 (0–2491)	34 (0–3960)			
Vegetables, g/day	82 ± 79	89 ± 92	1.00	1.00–1.00	0.138
	64 (0–828)	66 (0–4705)			
Fruits, g/day	154 ± 156	146 ± 170	1.00	1.00–1.00	0.342
	119 (0–1275)	108 (0–9468)			
Fermented foods, g/day	116 ± 140	121 ± 159	1.00	1.00–1.00	0.518
	84 (0–1153)	84 (0–3171)			
Estimated folic acid from daily meals, μg/day	260 ± 161	259 ± 163	1.00	1.00–1.00	0.947
	223 (17–1411)	230 (3–9253)			
**Supplementation during second and third trimesters**					
Folic acid					
Daily	54/341 (15.8)	19,467/89,138 (21.8)	Reference	–	< 0.01*
At least once a week	42/341 (12.3)	12,554/89,138 (14.1)	1.21	0.81–1.82	
At least once a month	28/341 (8.2)	6,605/89,138 (7.4)	1.53	0.97–2.44	
Never	217/341 (63.6)	50,512/89,138 (56.7)	1.55	1.15–2.08	
Zinc	8/338 (2.4)	4,052/88,910 (4.6)	0.51	0.25–1.02	0.058
Eicosapentaenoic acid	6/339 (1.8)	1,289/88,789 (1.5)	1.22	0.54–2.75	0.626
Docosahexaenoic acid	8/340 (2.4)	2,507/88,843 (2.8)	0.83	0.41–1.68	0.603
* Lactobacillus*-fermented beverage	176/341 (51.6)	47,021/89,048 (52.8)	0.95	0.77–1.18	0.660
**Habits**					
Smoking during pregnancy	17/335 (5.1)	4,247/88,178 (4.8)	1.06	0.65–1.72	0.826
Drinking during pregnancy	42/336 (12.5)	9,978/88,257 (11.3)	1.12	0.81–1.55	0.490

Data are presented as n (%), mean ± standard deviation, or median (range).

*KD* Kawasaki disease, *95% CI* 95% confidence interval.

*P-value for trend.

**Table 3:** Maternal perinatal backgrounds and child characteristics of the study population.

Variables	KD group (n = 343)	Control group (n = 90,143)	Odds ratio	95% CI	P value
Maternal age at birth, years	31 ± 5	31 ± 5	0.99	0.80–1.22	0.892
	31 (20–45)	31 (14–50)			
**Perinatal complications**					
Threatened abortion/premature labor	98/343 (28.6)	24,352/89,952 (27.1)	1.08	0.85–1.36	0.533
Gestational diabetes	6/343 (1.7)	2,450/89,952 (2.7)	0.64	0.28–1.43	0.272
Gestational hypertension	11/343 (3.2)	2,849/89,952 (3.2)	1.01	0.55–1.85	0.967
Premature rupture of membranes	36/343 (10.5)	7,507/89,952 (8.3)	1.29	0.91–1.82	0.152
**Mode of delivery**					
Cesarean section	59/342 (17.3)	17,819/89,728 (19.9)	0.84	0.64–1.11	0.228
**Child characteristics**					
Preterm birth (≤ 36 weeks)	15/343 (4.4)	4,830/89,952 (5.4)	0.81	0.48–1.35	0.415
Male	187/343 (54.5)	46,210/90,141 (51.3)	1.14	0.92–1.41	0.229
Birth weight, g	3,021 ± 409	3,011 ± 428	0.99	0.97–1.02	0.664
	3,020 (914–4,304)	3,020 (398–5,214)			
Neonatal jaundice with treatment	53/337 (15.7)	13,697/87,371 (15.7)	1.00	0.75–1.35	0.980
Breastfeeding only	142/340 (41.8)	37,958/89,598 (42.4)	0.98	0.79–1.21	0.823
Weight gain per day, g/day	39 ± 11	39 ± 12	0.98	0.89–1.07	0.612
	39 (3–74)	39 (9–89)			
Presence of siblings of child	224/341 (65.7)	51,962/89,605 (58.0)	1.39	1.11–1.74	< 0.01

Data are presented as n (%), mean ± standard deviation, or median (range).

*KD* Kawasaki disease, *95% CI* 95% confidence interval.

Table 4 shows the results of a multivariable logistic regression analysis. Maternal thyroid disease during pregnancy, insufficient intake of folic acid during pregnancy, and presence of siblings of the affected child were risk factors associated with KD onset in infancy.

**Table 4:** Association between variables and onset of Kawasaki disease in infancy.

Variables	Adjusted odds ratio	95% CI	P value
Maternal thyroid disease during pregnancy	2.03	1.04–3.94	0.037
Insufficient intake of folic acid supplementation during pregnancy	1.37	1.08–1.74	0.009
Male	1.15	0.93–1.42	0.200
Presence of siblings of child	1.33	1.06–1.67	0.014

*KD* Kawasaki disease, *95% CI* 95% confidence interval.

## Discussion

We conducted a comprehensive survey of demographic factors, medical and obstetric history, physical and mental health, lifestyle, environmental exposure, dwelling conditions, and socioeconomic status from the first trimester to 12 months after birth in the JECS nationwide birth cohort. Insufficient intake of folic acid during pregnancy, maternal complications of thyroid disease during pregnancy, and presence of siblings of the affected child were associated with KD in infancy. A novel finding in this study is the association between folic acid supplementation during pregnancy and the risk of KD. Folic acid supplementation during pregnancy reduces the risk of congenital malformations, such as neural tube defects^27,28^; it acts as a cofactor in the nucleotide biosynthesis pathway and methylation reaction system^29^. Amarasekera et al.^30^ reported that the neonatal cord blood concentration of folic acid is positively correlated with the maternal blood concentration of folic acid. In addition, they found that maternal folic acid supplementation upregulated DNA methylation and genomic imprinting in the CD4-positive cells of offspring^30^. Another study showed that folic acid suppresses gene expression of inflammatory cytokines, such as interleukin-1β, tumor necrosis factor-α, and monocyte chemoattractant protein-1 in lipopolysaccharide-activated macrophages^31^. These inflammatory cytokines play essential roles in the pathophysiology of KD. Therefore, we speculated that sufficient exposure to folic acid in utero might affect the development of immunological function in offspring and consequently decrease the risk of KD onset. Because the estimated folic acid from daily meals was not different between the KD group and the control group, further study is necessary to clarify the mechanism by which onset of KD in early infancy can be prevented by maternal folic acid supplementation.

We found that maternal complications of thyroid disease during pregnancy were associated with a significantly increased risk of KD onset in infancy. Belkaibech et al.^23^ reported that maternal autoimmune diseases, particularly autoimmune thyroid disease, are risk factors for KD in offspring. They reported that the risk of KD onset at 1 year of age was 10.98-fold higher in the presence of maternal autoimmune thyroiditis (hazard ratio, 10.98; 95% CI 9.42–12.81)^23^. In our study, the risk of KD onset in infancy was 2.03-fold higher in the presence of maternal thyroid disease (odds ratio, 2.03; 95% CI 1.04–3.94). We believe that this difference may be influenced by the inclusion of all thyroid diseases in our study; we did not limit our participants to those with autoimmune thyroiditis. Concerning pathophysiology, Svensson et al.^32^ reported increased production of inflammatory cytokines and abnormal lymphocyte subsets in the cord blood of offspring with maternal autoimmune thyroiditis. We speculate that these immunological changes in offspring may be related to KD onset in infancy. Notably, however, the number of mothers at risk was relatively low in both studies; there were six in the study by Belkaibech et al.^23^ and nine in ours. Under these conditions, generalizability of the study results has not been fully assured because of a lack of statistical power. Further study is required to investigate the association between maternal thyroid disease and KD onset in offspring.

The presence of siblings significantly increased the risk of KD onset in infancy. However, whether KD is induced by infectious disease remains controversial. Various infectious agents, such as group A streptococci, *S. aureus*, *Y. pseudotuberculosis*, respiratory viruses, Epstein-Barr virus, and human adenovirus have been reported as triggers for KD onset^5–10^. Infants with siblings may be at a higher risk of exposure to infectious agents compared with infants without siblings^33,34^. Therefore, we speculate that infants with siblings are at an increased risk of KD onset. Unfortunately, we did not collect data regarding the number, timing, and pathogens of infectious episodes in this study; therefore, we cannot verify whether infants with siblings are at a higher risk of infection compared with infants without siblings.

Previous large cohort studies have revealed that a higher socioeconomic status and maternal smoking in early childhood increase the risk of KD onset^20,21^. Our study showed no significant association between these factors and KD onset. We investigated maternal smoking habits during pregnancy, whereas Yorifuji et al.^20^ investigated maternal smoking habits at 6 months of age. Differences in the timing of smoking made it difficult to compare the two studies. The income categories used in our study and previous studies were similar. In a previous study, only one income category (> 10 million JPY) was associated with a significantly increased risk of KD; there were no differences in the other income categories^21^. The number of people in this category was small, and significant differences should be interpreted with caution.

This study has several limitations. First, the JECS includes only 15 regions in Japan; therefore, this study might include sampling bias. However, the prevalence of KD onset in infancy is similar to that in a Japanese nationwide study (317 per 100,000 births from 2011 to 2014)^35,36^. Therefore, we believe that the influence of sampling bias in this study is minimal. Second, approximately 10,000 children dropped out of the JECS. This attrition may have had an impact on the exact outcome. Third, approximately 50% of fathers in JECS did not complete the questionnaires regarding paternal backgrounds and exposures. Fourth, we did not obtain details on the frequency or dose of supplementation during pregnancy. Therefore, we could not study the causal effect between the dose of folic acid supplementation and KD onset in this study. However, from the perspective of habituation, we have shown that daily intake of folic acid might reduce the risk of KD onset. Additional analyses are needed to explore the true causal relationship between folic acid supplementation and KD onset. Finally, in this study, we analyzed the risk of KD onset up to 1 year of age because the risk of KD onset after 1 year of age could not be estimated from the JECS database. In the future, we intend to conduct an analysis to estimate the time-dependent risk of KD onset in children up to 5 years of age.

In conclusion, we found that insufficient intake of folic acid during pregnancy, maternal complications of thyroid disease during pregnancy, and presence of siblings of the affected child were independent risk factors associated with KD onset in infancy. Further well-designed studies are needed to determine the causal relationship of these exposures to KD onset.

## Methods

### Study design, settings, and participants

In the JECS, participants were recruited across 15 regional centers from January 2011 to March 2014. The precise study design was published in 2014^24^. Briefly, pregnant women in the first trimester and their partners were asked to participate in the survey. The target sample number of the JECS was set at 100,000, which is equivalent to half the number of births in the study area. We obtained data on exposures from pregnant women and their partners using self-administered questionnaires in the first trimester; second or third trimester; and 1, 6, and 12 months after birth. The five priority outcomes of the JECS were reproduction and pregnancy complications, congenital anomalies, neuropsychiatric disorders, allergies and immune system deficiencies, and metabolic and endocrine system disorders, including KD^24^. The regular self-administered questionnaires also included items regarding the development of these outcomes. For children with KD onset, a detailed survey of KD (KD-specific questionnaire) was also sent to co-operating healthcare providers that treated participants. In this study, we excluded children who were miscarried or stillborn and those whose birth records were unavailable. We also excluded live-birth children whose parents did not respond to the questionnaire when their child was 6 months old and at 1 year of age. Finally, we excluded children whose parents stated that their child had no history of KD at 6 months but did not respond to the questionnaire when their child was 1 year of age.

The JECS was performed in accordance with the Declaration of Helsinki and the Japanese Ethical Guidelines for Epidemiological Research published by the Ministry of Education, Culture, Sports, Science and Technology and the Ministry of Health, Labour and Welfare, Japan. The JECS protocol was approved by the Japanese Ministry of the Environment’s Institutional Review Board on Epidemiological Studies and the ethics committees of all participating institutions (Yokohama City University, National Center for Child Health and Development, Kyoto University, Nagoya City University, Hokkaido University, Tohoku University, Fukushima Medical University, Chiba University, University of Yamanashi, University of Toyama, Osaka University, Hyogo College of Medicine, Tottori University, Kochi University, University of Occupational and Environmental Health, Kumamoto University and National Institute for Environmental Studies). In this study, we used the dataset jecs-ta-2019030-qsn, which was released in October 2019. All participants provided written informed consent. The JECS was registered in the UMIN Clinical Trials Registry (UMIN000030786).

### Variables

We obtained maternal information from the questionnaires at registration (the first trimester), during the second/third trimester, 1 month after birth, 6 months after birth, and 1 year after birth. We also obtained paternal information from the questionnaires at registration and information from doctors at registration (the first trimester), birth, and 1 month after birth. From each questionnaire in the first trimester, we obtained the maternal pre-pregnancy height and weight, body mass index (weight [kg]/height squared [m]^2^), and medical history regarding KD, allergic disease, neurological/psychiatric disease, diabetes mellitus, and cancer. We also obtained the paternal medical history regarding KD and allergic disease, the method of pregnancy (spontaneous pregnancy), and the presence of siblings of the participating child. From each questionnaire in the second/third trimester, we obtained the maternal dietary intake, use of supplements, and socioeconomic status. Items related to diet included beans (g/day), green and yellow vegetables (g/day), fruits (g/day), fermented foods (cheese, yogurt, nattō, and *Lactobacillus*-fermented beverages) (g/day), and folic acid (µg/day)^37^. Items related to the use of supplements included folic acid, zinc, eicosapentaenoic acid, docosahexaenoic acid, and *Lactobacillus*-fermented beverages. We categorized zinc, eicosapentaenoic acid, docosahexaenoic acid, and *Lactobacillus*-fermented beverages as having been consumed at least once a month, regardless of frequency. Meanwhile, we divided folic acid into four categories on the basis of the frequency of intake: daily, at least once a week, at least once a month, and never. Items regarding socioeconomic status included the mother’s and father’s education history and household income. We divided education history into three categories: junior high or high school graduate, technical junior college or technical/vocational college graduate or associate degree, and bachelor’s degree or beyond. Considering that the relative poverty line for a family was approximately 2 million JPY and the median household income was approximately 4 million JPY in Japan from 2010 to 2018, we divided household income into five categories: poor (< 2 million JPY), lower-middle (2 to < 4 million JPY), middle (4 to < 6 million JPY), higher-middle (6 to < 10 million JPY), and high (≥ 10 million JPY). We collected information on maternal smoking and drinking at two time points during pregnancy: in the first trimester and the second/third trimester. We defined mothers as having a smoking/drinking habit if they answered smoking/drinking at either or both time points. After delivery, we obtained the maternal age at the child’s birth, maternal complications during the pregnancy (thyroid disease, diabetes mellitus), perinatal complications (threatened abortion/premature labor, gestational diabetes mellitus, gestational hypertension, premature membrane rupture), mode of delivery (cesarean section), preterm birth (< 36 weeks), sex of the child (male), and weight of the child at birth. Similarly, we obtained data on neonatal jaundice requiring treatment, nutritional methods (breastfeeding only), and weight gain per day up to 1 month of age.

### Primary outcome

The primary outcome was KD onset from infancy to 1 year of age. We defined children with KD as those whose mothers checked “have a history of KD” on the questionnaire at 6 months and/or 1 year of age. KD was diagnosed using the diagnostic guidelines of Kawasaki disease, 5th edition^38^. We also obtained information on the age at onset, diagnostic certainty, treatment details, effect of initial intravenous immunoglobulin, presence of coronary artery lesions in the acute phase, and family history of KD from the KD-specific questionnaire. The KD-specific questionnaire was completed by the child’s physician if their parents answered that their child had a history of KD.

### Statistical analysis

Data are presented as mean ± standard deviation or median with range for continuous variables and percentages for categorical variables. A two-tailed P value of < 0.05 was considered statistically significant. We conducted all statistical analyses using SAS software version 9.4 (SAS Institute, Cary, NC, USA).

Through a literature review, we identified 41 potential risk factors for KD, including demographic factors, medical and obstetric history, physical and mental health, lifestyle, environmental exposure, dwelling conditions, and socioeconomic status. These variables were screened through univariate logistic regression analysis and trend tests of logistic regression for ordinal variables. We performed a multivariate logistic regression analysis using two criteria for variable selection: (1) the variables that were considered to be significantly associated with KD onset with univariate analysis (these variables did not include the paternal medical history because of a large amount of missing data); (2) the variables that seemed clinically important or were reported when considering the etiology of KD. With multivariate analysis, ordinal variables were dichotomized into categories: intake more than once a week and intake less than two or three times a month for clinical significance.
